# Detection of *Mycobacterium tuberculosis* DNA on the oral mucosa of tuberculosis patients

**DOI:** 10.1038/srep08668

**Published:** 2015-03-02

**Authors:** Rachel C. Wood, Angelique K. Luabeya, Kris M. Weigel, Alicia K. Wilbur, Lisa Jones-Engel, Mark Hatherill, Gerard A. Cangelosi

**Affiliations:** 1 Department of Environmental and Occupational Health Sciences, School of Public Health, University of Washington, Seattle, WA, USA; 2 South African Tuberculosis Vaccine Initiative (SATVI), School of Child & Adolescent Health, University of Cape Town, Cape Town, South Africa; 3 Evolutionary Emergence of Infectious Diseases Laboratory, National Primate Research Center, University of Washington, Seattle, WA, USA

## Abstract

Diagnosis of pulmonary tuberculosis (TB) usually includes laboratory analysis of sputum, a viscous material derived from deep in the airways of patients with active disease. As a diagnostic sample matrix, sputum can be difficult to collect and analyze by microbiological and molecular techniques. An alternative, less invasive sample matrix could greatly simplify TB diagnosis. We hypothesized that *Mycobacterium tuberculosis* cells or DNA accumulate on the oral epithelia of pulmonary TB patients, and can be collected and detected by using oral (buccal) swabs. To test this hypothesis, 3 swabs each were collected from 20 subjects with active pulmonary TB and from 20 healthy controls. Samples were tested by using a polymerase chain reaction (PCR) specific to the *M. tuberculosis* IS6110 insertion element. Eighteen out of 20 confirmed case subjects (90%) yielded at least 2 positive swabs. Healthy control samples were 100% negative. This case-control study supports past reports of *M. tuberculosis* DNA detection in oral swabs. Oral swab samples are non-invasive, non-viscous, and easy to collect with or without active TB symptoms. These characteristics may enable simpler and more active TB case finding strategies.

TB remains a significant global health problem^1^. The best available way to control transmission of *Mycobacterium tuberculosis* is to promptly identify and treat active TB cases. Diagnosis of pulmonary TB is usually done by microbiological, microscopic, or molecular analysis of patient sputum^2^. The need for sputum as a diagnostic sample is a limiting factor due to the challenges of collecting it from patients and to its complex composition. The viscosity of the material restricts test sensitivity, increases sample-to-sample heterogeneity, and increases costs and labor associated with testing. Moreover, sputum production (which requires coughing) is an occupational hazard for healthcare workers^3^. A sample that is easier, safer, and more uniform to collect and handle would simplify TB diagnosis by PCR.

The search for an alternative to sputum has been ongoing for years with limited success. A recent study^4^ applied a commercial PCR test for TB, the GeneXpert MTB/RIF, to a variety of nontraditional samples obtained from culture-confirmed, HIV negative, pulmonary TB patients. The samples included exhaled breath concentrate (EBC), saliva, blood, and urine. Sensitivities relative to culture ranged from 0% (EBC, 0/26) to 38.5% (saliva, 10/26), far below the 100% seen when the GeneXpert test was applied to sputum from the same patients^4^. Another study used Xpert to analyze stool samples from children with culture-confirmed pulmonary TB^5^. Among a small sample of HIV positive children, the sensitivity relative to culture was 80% (4/5); however, in the absence of HIV co-infection, sensitivity relative to culture was 33% (4/12). GeneXpert testing of sputum samples from these subjects was 65% sensitive relative to culture (11/17).

Previous studies have shown that *M. tuberculosis* DNA can be detected in oral (buccal) swabs from human and non-human primates^6^,^7^,^8^. These studies did not attempt to correlate *M. tuberculosis* DNA detection with laboratory- or clinically-confirmed TB disease in humans. Such benchmarks are needed to rule out artifacts including false positive PCR results due to field or laboratory contamination of samples. However, the concept appeared feasible, in part because *Mycobacterium* cells, like most bacteria, have evolved mechanisms to adhere to surfaces, including mammalian cells. *M. leprae* cells were shown to adhere to nasal and alveolar epithelial cells^9^, while *M. avium* cells were shown to adhere to the bronchial epithelium^10^ and to non-biological surfaces^11^,^12^. In nature, many environmental *Mycobacterium* species are more commonly associated with surfaces than with fluid matrices^13^. Based on these observations, we hypothesized that some bacilli that pass through the mouths of TB patients might accumulate on the oral epithelium and be detectable by analysis of oral swab samples. In order to confirm the disease-specificity of such observations, oral swab PCR (OSP) was conducted on swab samples collected from GeneXpert-confirmed TB patients as well as from healthy controls. This is the first rigorously controlled study to test the OSP concept.

## Results

### Case-control study design and population

In this case-control study, 20 newly diagnosed pulmonary TB patients in Worcester, South Africa were selected as cases and matched to 20 healthy control subjects in Seattle, WA, USA. Case and control subjects were matched on gender and 10-year age range. All TB case subjects were HIV-negative. Case subjects qualified for the study by virtue of positive sputum GeneXpert MTB/RIF^14^, and all of them were clinically diagnosed and treated for the disease.

Healthy control subjects were included in the study to test for false-positive PCR results arising from laboratory contamination. Given the novelty of the sampling method under investigation, we could not in advance exclude the possibility that healthy but recently-exposed people, or latently infected people, might occasionally yield positive OSP results. Therefore, Seattle was chosen for control subject recruitment because of its very low incidence of TB (5.5 cases per 100,000)^15^. All control subjects were confirmed healthy and unexposed to TB via questionnaire. The questionnaire determined control subject eligibility based on domestic risk factors including previous diagnosis of TB, previous positive PPD or Quantiferon-TB Gold test, travel to high TB burden countries, and time spent with domestic high-incidence populations.

Each group included 8 women and 12 men; all over the age 21. The median age of the women in the case group was 38, IQR (31–51) and 40, IQR (33–51) in the control group. The median age of the men in the case group was 45, IQR (35–56), and in the control group, it was 42, IQR (38–54).

### Sample acquisition and results of analysis

Three swabs were collected by the researchers from each subject on three separate days within a 7-day time span. All swabs were collected from the case subjects at least 1 day after they had received a positive diagnosis, and no more than 7 days post-initiation of treatment. Studies have shown that sputum loads of *M. tuberculosis* DNA do not change dramatically during this initial phase of treatment^16^.

Swabs were brushed along the inside of the subject's cheek for about 10 seconds (7–8 times) to collect cells and saliva. The head of each swab was ejected into a tube containing 500 μl of a sterile lysis buffer. Negative field samples (swabs briefly exposed to the air at the sampling site and put into lysis buffer) were also collected. Samples were stored frozen until analysis. Nucleic acid was extracted from samples and tested for *M. tuberculosis* DNA by using a non-nested quantitative PCR assay, based on the protocol described by Halse et al, 2010 targeting the IS6110 insertion element, which is unique to the *M. tuberculosis* complex^17^,^18^.

In a first-pass, partial volume analysis conducted on all samples, 10% of total extracted sample volume (5 μL) was analyzed. Case samples that were negative in the partial volume analysis were concentrated by ethanol precipitation and subjected to a second-pass, full volume analysis corresponding to 90% of total sample volume. To control for sample contamination during ethanol precipitation and suspension, full volume analysis was also applied to the corresponding healthy control samples.

Upon testing by OSP, 18 of the 20 case subjects (90%) yielded at least 2 swabs positive for *M. tuberculosis* DNA either in partial volume or full volume analyses (Table 1). Of the 60 swab samples collected from the case group, 44 (73.3%) were positive. All control subjects were negative in all 3 swab samples per subject, in both the partial volume and full volume analyses (Table 1). The positive partial volume results had Cq-values ranging from 28.0 to 41.5, while the positive full volume results had Cq-values ranging from 35.4 to 40.0. Negative results were defined by lack of amplification after 45 cycles. Procedural negative controls were also 100% negative.

Sputum smear (acid fast) microscopy results were available for 17 of the 20 case subjects, of which 10 (59%) were smear positive (Table 1). Sensitivity of OSP performance relative to GeneXpert MTB/RIF is summarized for all subjects and for sputum smear-positive subjects in Table 2. Specificity of OSP was 100% relative to the presumed disease-negative status of healthy controls.

### Analysis of factors associated with OSP negativity

Four subjects (numbers 3, 4, 6, and 16) were negative in all three swabs after partial volume analysis, and two of them (numbers 3 and 4) remained negative after full volume analysis (Table 1). Sputum smear negativity is often associated with reduced pathogen load and reduced clinical sensitivity of sputum-based molecular diagnostics, including the GeneXpert MTB/RIF^14^. However, smear negativity did not exhibit detectable correlation with OSP negativity within our sample (Fisher's exact test, p = 0.25 and 0.15 in partial volume and full volume analyses, respectively). Four of the 20 case subjects (numbers 3, 4, 6, and 14; Table 1) were being treated for diabetes mellitus, a known risk factor for TB^19^. Active treatment for this condition correlated with OSP-negativity (Fisher's exact test, p = 0.013 and 0.032 in partial volume and full volume analyses, respectively). No correlations were seen with other factors including gender and date of sample collection (not shown).

## Discussion

The results of this proof-of-concept study showed that *M. tuberculosis* DNA can be detected in the oral mucosa of the majority of adult patients with positive GeneXpert MTB/RIF results and clinically confirmed pulmonary TB. Detection of *M. tuberculosis* DNA in oral mucosa has been reported previously in human and non-human primates^6^,^7^,^8^, however the present study is the first to test and quantify this phenomenon relative to confirmed TB disease in humans, within a case-control study structure. The results appear to exclude artifactual findings resulting from sample contamination. They demonstrate that *M. tuberculosis* DNA is a common occurrence in these samples.

The results do not tell us whether positive OSP signals are associated with intact *M. tuberculosis* cells, free DNA, or both. Moreover, nothing is known about the biology of *M. tuberculosis* association with buccal cells. Bacilli regularly pass from lungs and airways through the mouths of TB patients. We conjecture that a portion of these bacilli bind to buccal cells, either specifically or non-specifically. There is no evidence that this association involves infection or mycobacterial growth within buccal cells. If the “on rate” of this association exceeds the “off rate”, then *M. tuberculosis* cells could accumulate in the oral mucosa and be detectable there even when there is no positive sputum in the mouth. This raises the future possibility of using OSP to diagnose TB in children or other patients who do not frequently produce positive sputum.

In the current proof-of-concept study conducted on HIV-negative, adult subjects, 18 of 20 case subjects (90%) yielded at least 2 positive swabs. Future improvements to sampling and analytical techniques could further improve the sensitivity of OSP. In contrast to other studies that measured the sensitivity of saliva, blood, urine, EBC, or stool analyses relative to culture^4^,^5^, the present analysis measured the sensitivity of OSP relative to GeneXpert results. Therefore, sensitivities are difficult to compare between studies. Nonetheless, OSP performed well relative to GeneXpert, when at least 2 swabs per patient were analyzed. If larger studies validate these findings, then OSP may be an advantageous sample technique for TB diagnosis and case finding. The buccal swab is very easy to collect, requiring just seconds and no specialized equipment or invasive techniques. Relative to sputum, buccal swab samples have smaller volumes, are more uniform in volume and composition, and are less viscous and heterogeneous. These characteristics may enable simpler and less expensive diagnostic approaches, especially in point-of-care strategies under development. Although the current study used a laboratory-based PCR as a detection method, the use of other detection methods including culture, microscopy, antigen detection, and POC nucleic acid testing devices, can be explored in future studies.

In addition to facilitating passive (diagnostic) TB case finding, OSP could enable more active TB case finding approaches among individuals who do not actively produce sputum. It may be an attractive alternative to invasive gastric lavage or sputum induction in younger children who cannot expectorate a sputum sample at request; and to bronchoscopy in persons with suspected TB who are unproductive of sputum.

Preliminary evidence, based on a limited sample, was obtained of an association between OSP negativity and ongoing treatment for diabetes mellitus. Because these patients were already under care for a condition that is known to increase risk of active TB, there is a possibility that their TB disease may have been detected at a relatively early stage with lower bacillary load. Consistent with this explanation, at least 3 of the 4 diabetes patients were sputum smear negative (no smear data were available for the fourth). Additionally, diabetes is associated with changes in oral mucosa. Diabetics sometimes exhibit reduced salivary flows and are more susceptible to oral infections, which could correlate with elevated loads of oral microbial flora, including native flora^20^,^21^. This could decrease opportunities for non-native *M. tuberculosis* cells to adhere to buccal cells where they would be detectable by OSP. However, the sample was too small to draw definitive conclusions about any of these factors.

Although the case and control groups differed significantly in the number of OSP-positive samples and subjects, additional studies with larger and more diverse patient populations are needed. The study had several additional limitations that must be addressed in follow-up studies. Only HIV-negative, adult TB case subjects were included. HIV status and age can affect bacillary load, which could influence OSP detection. GeneXpert-negative and culture-positive patients, who may have lower bacillary burdens than GeneXpert-positive patients, were not included in the study. Additionally, the case and control groups were not well matched in terms of ethnicity, location, and infectious disease exposure. A control group more similar to the case group would strengthen future studies. This study also did not address innate limitations of PCR-based testing, including the inability to detect certain drug resistance phenotypes and to differentiate viable from non-viable bacteria^22^. The study used combined clinical and laboratory (GeneXpert) diagnosis as a reference standard for positivity, rather than sputum culture. Finally, the study did not explore alternative buccal swab sample collection and analysis methods, which could further improve the sensitivity of the technique.

In conclusion, the results demonstrate that *M. tuberculosis* DNA can be detected with high frequency in the oral mucosa of adult patients with active pulmonary TB. OSP may have promise as an alternative to traditional sputum based testing for TB diagnosis. With further development of sample collection and analysis methods, OSP could simplify the molecular diagnosis of TB and potentially be used for active TB case finding, made possible by the simple, non-invasive nature of the sampling method. Faster identification and earlier treatment of active TB disease is vital to halt transmission and control the epidemic.

## Methods

### Study design & population

All methods were carried out in accordance with approved guidelines. In this proof-of-concept, case-control study, 20 newly diagnosed pulmonary TB patients in Worcester, South Africa were selected as cases and matched to 20 healthy control subjects in Seattle, WA, USA. Subjects in the case group were actively recruited through a pre-screening process using notification registers at TB clinics in Worcester, South Africa. The control subjects were passively recruited through flyers and email notifications.

Before swab collection, all TB case subjects were confirmed by positive sputum GeneXpert MTB/RIF and by HIV-negativity. All were clinically diagnosed TB cases who were prescribed treatment for the disease. The first 20 subjects that were eligible and provided informed consent were included in the study. All control subjects were confirmed healthy and TB unexposed via questionnaire. The questionnaire determined control subject eligibility based on domestic risk factors including previous diagnosis of TB, previous positive PPD or Quantiferon-TB Gold test, travel to high TB burden countries, time spent with homeless populations, time spent with immigrant groups, and time spent on American Indian reservations. Control subjects were matched to the case subjects on gender and 10-year age range.

### Sample acquisition

The researchers collected 3 swabs from each subject on 3 separate days, within a period of 7 days. Swabs were collected from the case subjects immediately after a positive diagnosis, and before they had been on treatment for 7 days.

The swabs (OmniSwab, Whatman) were brushed along the inside of the subject's cheek for about 10 seconds (7–8 times) to collect cells and saliva. The head of each swab was ejected into a tube containing 500 μl of a sterile lysis buffer consisting of 50 mM Tris pH 8.0, 50 mM EDTA, 50 mM sucrose, 100 mM NaCl, and 1% SDS at room temperature^8^. Negative field samples (swabs briefly exposed to the air at the sampling site and put into lysis buffer) were collected with every case sample and with every 3 control samples. Samples were stored at −80°C and case samples were shipped from Worcester, South Africa to Seattle, WA, USA on dry ice. All qPCR analyses were conducted in Seattle.

### Laboratory analysis

Prior to opening the tube and initiating DNA extraction, each sample of 500 μl buffer and swab was heated to 95°C for 10 minutes to inactivate and partially disrupt *M. tuberculosis* cells. DNA extraction of the full 500 μl volume was completed using the QIAGEN QIAamp DNA mini kit spin column protocol. The first step of the QIAamp protocol was omitted because it called for the addition of a buffer, which was unnecessary as the swabs were already in the lysis buffer. To begin the extraction, materials in the samples were suspended in the lysis buffer through vortexing and agitation. Subsequent steps followed the manufacturer's instructions. The final step was altered so that the samples were incubated at 42°C for 3 minutes and at room temperature for 2 minutes before elution with 50 μl of AE buffer. This increased the contact time of the buffer on the silica in order to maximize the yield. At least one negative extraction sample (lysis buffer, no swab) was extracted alongside every batch of samples, which ranged in size from 5 to 12 samples.

In the first-pass (partial volume) analysis, aliquots (5 μl) of the 50 μl DNA eluates were analyzed using a probe-based qPCR protocol targeting a 74 bp amplicon on the IS6110 insertion sequence^17^,^18^. qPCR analysis was completed on a 25 μl reaction mixture that consisted of 1× LightCycler Master Mix (Roche), 2.0 mM MgCl_2_, 0.45 μM forward primer, 1.35 μM reverse primer, 0.25 μM FAM/MGBNFQ probe, 7.750 μl H_2_O, and 5 μl DNA. qPCR was performed on the Applied Biosystems StepOnePlus Real-Time PCR system using the following reaction protocol: initial incubation at 95°C for 10 min and 45 cycles of 95°C for 15 seconds (denaturation) and 60°C for 1 minute (annealing/extension). At least 2 negative PCR controls (5 μl of H_2_O instead of DNA) were included in each run to ensure no contamination occurred during PCR setup.

In the second-pass (full volume) analysis, eluates of case samples that tested negative in partial volume analysis were concentrated approximately 9-fold by ethanol precipitation of the 45 μl eluate volume that remained after the partial volume analysis. Precipitates were resuspended in 5 μl of AE buffer. Negative precipitation control samples (45 μl of AE buffer, no swab) were precipitated alongside the samples. The same PCR protocol as described above was used to reanalyze the samples. Full volume analysis was also applied to healthy control samples matching the case samples that converted to positive upon full volume analysis.

### Statistical analyses

STATA 12.1 and VassarStats were used for the statistical analyses, which include the Fisher's exact test and sensitivity and specificity calculations.

### Human subject protection

All subjects were compensated for their time and participation, and written informed consent was collected from each subject. All study protocols were approved by by the Human Research Ethics Committee of the Faculty of Health Sciences, University of Cape Town (HREC Ref No. 266/2013) and the Human Subjects Division at the University of Washington approved this study (HSD Protocol No. 45151).

## Author Contributions

R.W., K.W. and A.L. planned and performed experiments, interpreted results, and co-wrote the manuscript. L.J.E., M.H. and G.C. planned experiments, interpreted results, and co-wrote the manuscript. A.W. planned experiments and interpreted results. R.C.W. and A.K.L. contributed equally to this work, as did M.H. and G.A.C.

## Figures and Tables

**Table 1:** Data on sputum smear results, Diabetes mellitus, and OSP results

Subject pair	Xpert Positive Cases^1^					Matched healthy controls		
	Sputum Smear Results	DM^2^	OSP^3^, Sample A	OSP, Sample B	OSP, Sample C	OSP, Sample A	OSP, Sample B	OSP, Sample C
1	+		Negative*	+*	+	Negative	Negative*	Negative
2	ND^4^		Negative*	+	+	Negative	Negative	Negative
3	Negative	+	Negative*	Negative*	Negative*	Negative	Negative	Negative
4	Negative	+	Negative*	Negative*	Negative*	Negative	Negative	Negative
5	Negative		+	+	+	Negative	Negative	Negative
6	Negative	+	+*	Negative*	+*	Negative*	Negative	Negative*
7	+++		+	+	+	Negative	Negative	Negative
8	+++		+	+	+	Negative	Negative	Negative
9	+++		+	+	+	Negative	Negative	Negative
10	+		+	+	Negative*	Negative	Negative	Negative
11	+++		+	+	+	Negative	Negative	Negative
12	++		+*	+	Negative*	Negative*	Negative	Negative
13	Negative		Negative*	+	+	Negative	Negative	Negative
14	ND	+	+	+	+	Negative	Negative	Negative
15	Negative		Negative*	+	+	Negative	Negative	Negative
16	+++		+*	+*	+*	Negative*	Negative*	Negative*
17	Negative		Negative*	+*	+	Negative	Negative*	Negative
18	ND		+	Negative*	+	Negative	Negative	Negative
19	+++		+	+	+	Negative	Negative	Negative
20	++		Negative*	+	+	Negative	Negative	Negative

^1^All 20 case subjects were positive by GeneXpert testing and clinical assessment, and were subsequently treated for tuberculosis.

^2^DM, Diabetes mellitus. Cases 3, 4, 6, and 14 were in treatment for DM. Other cases were presumed DM-negative.

^3^For OSP, results of partial volume analysis are shown except values marked by asterisk (*), which are the full volume analysis conducted after partial results were negative. Negative results had Cq > 45.

^4^ND, not determined.

**Table 2:** OSP sensitivity and specificity relative to GeneXpert MTB/RIF among all subjects and among smear positive subjects

		Sensitivity OSP positive/total Xpert positive, % (95% CI)	Specificity OSP negative/total Xpert negative, % (95% CI)
**All subjects**	By subject	18/20, 90.0% (66.9–98.2)	20/20, 100.0% (80.0–100)
	By swab	44/60, 73.3% (60.1–83.5)	60/60, 100.0% (92.5–100)
**Smear + subjects**	By subject	10/10, 100.0% (65.5–100)	10/10, 100.0% (65.5–100)
	By swab	26/30, 86.7% (68.4–95.6)	30/30, 100.0% (85.9–100)
